# The causal role of breakfast in energy balance and health: a randomized controlled trial in obese adults^1^,^2^

**DOI:** 10.3945/ajcn.115.122044

**Published:** 2016-02-10

**Authors:** Enhad A Chowdhury, Judith D Richardson, Geoffrey D Holman, Kostas Tsintzas, Dylan Thompson, James A Betts

**Affiliations:** 3 Departments of Health and; 4 Biology and Biochemistry, University of Bath, Bath, United Kingdom; and; 5 School of Life Sciences, Queen’s Medical Centre, University of Nottingham, Nottingham, United Kingdom

**Keywords:** breakfast, energy balance, fasting, physical activity, energy intake, appetite regulation, obesity, metabolism

## Abstract

**Background:** The causal nature of associations between breakfast and health remain unclear in obese individuals.

**Objective:** We sought to conduct a randomized controlled trial to examine causal links between breakfast habits and components of energy balance in free-living obese humans.

**Design:** The Bath Breakfast Project is a randomized controlled trial with repeated measures at baseline and follow-up among a cohort in South West England aged 21–60 y with dual-energy X-ray absorptiometry–derived fat mass indexes of ≥13 kg/m^2^ for women (*n* = 15) and ≥9 kg/m^2^ for men (*n* = 8). Components of energy balance (resting metabolic rate, physical activity thermogenesis, diet-induced thermogenesis, and energy intake) were measured under free-living conditions with random allocation to daily breakfast (≥700 kcal before 1100) or extended fasting (0 kcal until 1200) for 6 wk, with baseline and follow-up measures of health markers (e.g., hematology/adipose biopsies).

**Results:** Breakfast resulted in greater physical activity thermogenesis during the morning than when fasting during that period (difference: 188 kcal/d; 95% CI: 40, 335) but without any consistent effect on 24-h physical activity thermogenesis (difference: 272 kcal/d; 95% CI: −254, 798). Energy intake was not significantly greater with breakfast than fasting (difference: 338 kcal/d; 95% CI: −313, 988). Body mass increased across both groups over time but with no treatment effects on body composition or any change in resting metabolic rate (stable within 8 kcal/d). Metabolic/cardiovascular health also did not respond to treatments, except for a reduced insulinemic response to an oral-glucose-tolerance test over time with daily breakfast relative to an increase with daily fasting (*P* = 0.05).

**Conclusions:** In obese adults, daily breakfast leads to greater physical activity during the morning, whereas morning fasting results in partial dietary compensation (i.e., greater energy intake) later in the day. There were no differences between groups in weight change and most health outcomes, but insulin sensitivity increased with breakfast relative to fasting. This trial was registered at www.isrctn.org as ISRCTN31521726.

## INTRODUCTION

Despite strong public belief regarding the role of regular breakfast in human health (1), most evidence linking the omission of breakfast with negative health outcomes is based on cross-sectional associations and prospective cohort studies (2–6). Nonetheless, randomized controlled trials in free-living adults have begun to question the causal nature of these links between breakfast habits, components of energy balance, and health (7–15).

Many experimenters have compared different breakfast types, informing conclusions regarding the effects of size or composition (8, 10, 16–19), as opposed to the fundamental contrast between the presence or absence of morning feeding. Trials that have investigated extended morning fasting represent a range of experimental approaches—from translating the acute metabolic/behavioral responses to fasting on a given morning or number of days (7, 12, 13, 20–23) to studying the health effects of skipping breakfast for weeks (11, 14) and ultimately the effect on body mass of recommendations to skip breakfast for 4 mo (9). Our recent work complemented these studies by examining the effects of extended morning fasting for 6 wk on all components of energy balance and health in lean individuals (15). In particular, we took advantage of free-living metabolic and behavioral monitoring to provide the first report to our knowledge of physical activity thermogenesis in response to breakfast, revealing greater energy expenditure relative to morning fasting but with little evidence of compensatory feeding later in the day.

It is important to note that it cannot be assumed that individual components of energy balance would respond similarly in the obese. Indeed, cross-sectional evidence in adolescent girls questions whether the relation between breakfast habits and physical activity may be moderated by adiposity (24). Obese individuals are more receptive to external cues to eat (25, 26) and display delayed satiation (27), possibly in part because of reduced concentrations of the satiety hormone peptide YY (28) and limited feeding-induced suppression of the orexigenic hormone ghrelin (29, 30).

Effective weight loss strategies are particularly relevant for obese individuals, yet such strategies can potentially be misguided if informed solely by the positive cross-sectional association between breakfast omission and obesity (5) and reports that, for example, 78% of successful dieters consume breakfast daily (31). Emerging trials in the overweight/obese are crucial for informing evidence-based weight loss strategies (9, 11, 32), with the largest of these trials recently demonstrating no effect of breakfast recommendations on weight loss (9). However, specific components of energy balance such as physical activity/exercise can affect disease and mortality risk independent of a net energy surplus/deficit or changes in adiposity (33, 34). Our recent report of daily breakfast compared with fasting affecting physical activity energy expenditure occurred without noteworthy changes in body mass in lean individuals (15). Considering the associations between breakfast omission and disease risk (2–5), it is prudent to establish whether these observations are causal and attributable to mechanisms independent of body mass.

This randomized controlled trial examined a 6-wk period of breakfast consumption or morning fasting and used assessments of energy balance and health in an obese population. Consistent with our previous report in lean individuals, we hypothesized that physical activity thermogenesis would be lower when fasting during the morning but that—unlike in lean individuals—extended morning fasting would also result in compensatory energy intake.

## METHODS

### Experimental design

The Bath Breakfast Project (ISRCTN31521726) is a randomized controlled trial that compares the effects of daily breakfast consumption relative to extended morning fasting on energy balance and human health. The procedures followed were in accordance with the protocol approved by the National Health Service South West 3 Research Ethics Committee (10/H0106/13). This protocol has since been published in full (15, 35), with trial enrollment, baseline/eligibility testing, allocation, and follow-up all conducted in accordance with Consolidated Standards of Reporting Trials guidelines (36). (See **Supplemental Figure 1** for a Consolidated Standards of Reporting Trials flow diagram and **Supplemental Figures 2** and **3** for the precise details of this protocol and the rationale for our approach/methods, respectively.) As justified in our published protocol (35), ∼14 people in each treatment group were deemed necessary to confer a 90% probability of detecting an increase in physical activity energy expenditure of 646 kcal with use of a 2-tailed *t* test with an α level of 0.05. Herein we report data for the obese cohort from the Bath Breakfast Project, classified according to dual-energy X-ray absorptiometry-derived fat mass indexes of ≥13 kg/m^2^ for women and ≥9 kg/m^2^ for men (37), for whom recruitment and follow-up lasted from 28 August 2010 to 24 May 2013. Study participants were recruited from local advertising, and invitations to participate were distributed to potentially eligible individuals via local general practice surgeries. Participating individuals did not receive any payment apart from any travel costs incurred for attending laboratory visits and all met the following inclusion criteria: aged 21–60 y; record of regular menstrual cycle/contraceptive use (if relevant); no anticipated changes in diet and/or physical activity habits during the study period; weight stable (within 2% over past 6 mo); nonshift workers; not pregnant or breastfeeding; and free from any other condition or behavior deemed either to pose undue personal risk or introduce bias into the experiment.

Baseline demographic and anthropometric characteristics of those who completed the trial are presented in **Table 1**. This cohort completed intensive laboratory-based assessments at baseline to determine their resting metabolic rate (via indirect calorimetry from gaseous exchange) and anthropometric characteristics, i.e., dual-energy X-ray absorptiometry (Hologic Discovery W); waist and hip circumference (midpoint between the lowest rib and iliac crest and the widest gluteal girth, respectively); and sagittal abdominal height (with use of a Holtain-Kahn caliper at the iliac crest). While participants remained in a 10-h overnight fast (0900 ± 1 h), a 15-mL blood sample was drawn from an antecubital vein via an indwelling cannula to determine concentrations of key systemic metabolites/hormones via commercially available spectrophotometric assays (HDL/LDL cholesterol, triacylglycerol, nonesterified fatty acids, glucose, and C-reactive protein from Randox Laboratories) and ELISAs [IL-6, leptin, and adiponectin from R&D Systems; triiodothyronine (free-T3) and thyroxine (free-T4) from Alpco Diagnostics; total and acylated ghrelin from Bertin Pharma; peptide YY and active glucagon-like peptide-1 from Millipore; and insulin from Mercodia]. A small (∼1 g) subcutaneous adipose tissue biopsy was then sampled from the abdomen to provide estimates of tissue-specific insulin action (i.e., insulin-stimulated [U-^14^C]d-glucose uptake). Participants then undertook an oral-glucose-tolerance test (OGTT) that involved ingesting 75 g glucose polymer in Polycal solution (Nutricia) with 5 mL arterialized venous blood samples drawn at baseline and every 15 min for 2 h after glucose ingestion from a separate cannula fitted retrograde to a dorsal vein on the back of the hand after warming for at least 15 min in a sealed box (Medical Engineering Unit, University of Nottingham) containing static air at 55°C (38).

**TABLE 1:** Baseline demographic and anthropometric characteristics and changes at follow-up^1^

	All participants (*n* = 23)		Breakfast group (*n* = 11)		Fasting group (*n* = 12)	
	Baseline	Change from baseline	Baseline	Change from baseline	Baseline	Change from baseline
Age, y	44 ± 10	—	44 ± 10	—	44 ± 10	—
Women, *n* (%)	15 (65)	—	7 (64)	—	8 (67)	—
Frequent habitual breakfast consumer,^2^ *n* (%)	14 (61)	—	7 (64)	—	7 (58)	—
Anthropometric characteristics						
Height, m	1.70 ± 0.10	—	1.71 ± 0.09	—	1.69 ± 0.11	—
BMI, kg/m^2^	33.7 ± 4.9	0.20 (0.02, 0.38)*	35.4 ± 6.1	0.33 (0.08, 0.58)	31.9 ± 2.3	0.07 (−0.19, 0.34)
Fat mass index (DXA),^3^ kg/m^2^						
All	13.3 ± 4.0	0.18 (−0.16, 0.52)	14.8 ± 5.0	0.16 (−0.55, 0.87)	12.0 ± 2.3	0.20 (−0.11, 0.52)
Women	15.1 ± 3.8	0.23 (−0.25, 0.71)	16.9 ± 4.5	0.21 (−0.84, 1.25)	13.2 ± 1.8	0.25 (−0.18, 0.69)
Men	9.8 ± 1.0	0.09 (−0.45, 0.63)	9.9 ± 1.4	0.06 (−1.82, 1.94)	9.8 ± 0.8	0.12 (−0.75, 0.99)
% Body fat (DXA)						
All	40.0 ± 7.5	0.44 (−0.49, 1.36)	42.6 ± 8.8	0.31 (−1.58, 2.19)	37.7 ± 5.6	0.55 (−0.39, 1.50)
Women	43.4 ± 6.1	0.55 (−0.59, 1.68)	46.9 ± 6.3	0.50 (−2.10, 3.11)	40.3 ± 4.1	0.58 (−0.47, 1.63)
Men	32.0 ± 2.3	−0.00 (−1.87, 1.86)	32.5 ± 3.4	−0.15 (−6.72, 6.42)	31.6 ± 1.5	0.11 (−2.84, 3.06)
Waist circumference, cm	104 ± 11	0.1 (−1.3, 1.6)	106 ± 14	1.2 (−1.0, 3.5)	103 ± 7	−1.0 (−2.9, 0.9)
Waist:hip ratio	0.89 ± 0.09	−0.00 (−0.14, 0.13)	0.87 ± 0.10	0.01 (−0.01, 0.03)	0.91 ± 0.07	−0.01 (−0.03, 0.00)
Sagittal abdominal diameter, cm	25.8 ± 2.7	−0.2 (−0.6, 0.2)	26.4 ± 3.2	0.0 (−0.4, 0.4)	25.2 ± 2.0	−0.4 (−1.0, 0.2)
Body mass, kg	98.2 ± 19.2	0.6 (0.1, 1.1)*	103.9 ± 24.0	1.0 (0.2, 1.7)	92.4 ± 11.2	0.2 (−0.5, 1.0)
Lean tissue mass (DXA),^4^ kg	53.6 ± 9.2	−0.00 (−1.0, 1.0)	52.5 ± 7.0	0.2 (−1.7, 2.1)	54.7 ± 11.0	−0.2 (−1.3, 0.8)
Adipose tissue mass (DXA), kg	37.8 ± 9.7	0.6 (−0.4, 1.5)	41.8 ± 12.8	0.5 (−1.4, 2.5)	34.1 ± 3.5	0.6 (−0.4, 1.5)

1 Data are means ± SDs at baseline, with Δ change and 95% CIs for the response within each group. *Significant response over time (*P* ≤ 0.05) as examined by 2-factor ANOVA. No variable differed significantly between groups at baseline, and there were no significant treatment × time interactions. DXA, dual-energy X-ray absorptiometry.

2 Defined as the ingestion of ≥50 kcal within 2 h of waking on most days of the week.

3 DXA-derived fat mass index obese ranges (37) = ≥13 kg/m^2^ (women) and ≥9 kg/m^2^ (men).

4 Lean tissue mass excludes bone mineral content.

All previously described measures were followed up 6 wk later, with free-living assessments of energy intake (estimated from directly weighed food diaries) and energy expenditure (combined heartrate/accelerometry) monitored by Actiheart (CamNtech) throughout the first and last weeks of intervention, along with continuous (5-min sampling interval) monitoring of interstitial glucose concentrations via a subcutaneous abdominal catheter (iPro; Medtronic) both to document chronic glycemic responses and to verify compliance (neither the continuous glucose monitoring system data nor food diary records provided any evidence of noncompliance to either intervention in any participants). During the intervening 4-wk period, participants were not monitored. For estimating energy intake, participants were provided with a set of food-weighing scales and trained by the experimenters during enrollment on how to appropriately record food intake. Packaging from preprepared items was kept by participants for analysis by the research team with use of the manufacturers’ information, with fresh foods input with use of Compeat Pro 5 dietary analysis software (Nutrition Systems). The Actiheart device for measuring energy expenditure is a chest-mounted device that was worn at all times apart from during water-based activities. This device uses heart rate and accelerometry combined in a branched equation model to estimate energy expenditure (39, 40) and has been shown to be a valid measure of energy expenditure in a variety of laboratory and free-living settings (41–43). Eumenorrheic women provided baseline samples 2 wk before the start of the 6-wk intervention so that follow-up samples could be acquired 3–10 d after the onset of menses (i.e., follicular phase). During the 6-wk intervention, participants were randomly assigned (1:1 allocation ratio) into either a group prescribed an energy intake of ≥700 kcal before 1100 daily, with at least half consumed within 2 h of waking (breakfast group), or a group to extend their overnight fast by abstaining from ingesting energy-providing nutrients (i.e., plain water only) until 1200 each day (fasting group). The randomization scheme was generated with use of a computer-based random-number generator and was stratified according to baseline breakfast habits (block size = 4), with frequent breakfast consumption defined as the ingestion of ≥50 kcal within 2 h of waking on most days of the week. The investigators who enrolled participants were unaware of these details and independently requested group assignments to prevent deciphering of the allocation sequence. Because of the self-administered nature of the treatments, it was not possible to blind participants to group allocation or to blind investigators for many outcomes that either required direct interaction with nonblinded participants (e.g., anthropometry and metabolic rate) or in which treatment allocation is immediately evident in the data (e.g., diet records and continuous glucose monitoring). These same investigators then also shared responsibility for completing various aspects of tissue and data analysis. The intervention was applied under free-living conditions. All other lifestyle choices were allowed to vary naturally. Compliance was confirmed via self-report and verified via continuous glucose monitoring. Four participants withdrew before baseline assessments (Supplemental Figure 1); data reported herein are therefore only for those individuals for whom baseline and follow-up measurements were available (i.e., a completers-only analysis).

### Data analysis

The primary outcome measures comprehensively assessed components of energy balance under free-living conditions, which were averaged from the first and last week of intervention and therefore expressed as simple summary statistics and analyzed with use of either paired or independent *t* tests for contrasts within and between groups, respectively. Secondary outcomes included regulatory/mechanistic data and markers of cardiovascular health and metabolic control at baseline and follow-up, for which treatment × time interactions were explored with use of a mixed-model ANOVA. Most variables in this experiment therefore involved a single comparison between 2 means and were not adjusted for multiple comparisons across the different variables reported herein (44). However, when multiple comparisons were made within a given variable (i.e., physical activity thermogenesis was partitioned according to intensity and time), a Holm-Bonferroni stepwise adjustment was applied to prevent type I error rate inflation (45). Statistical analyses were performed with use of SPSS version 22 (IBM), with statistical significance accepted at an α level of *P* ≤ 0.05. Values are means with SDs in text and tables and with SE bars in figures, with effects expressed as Δ change scores with 95% CIs.

## RESULTS

### Components of energy balance

#### Physical activity thermogenesis

**FIGURE 1:**
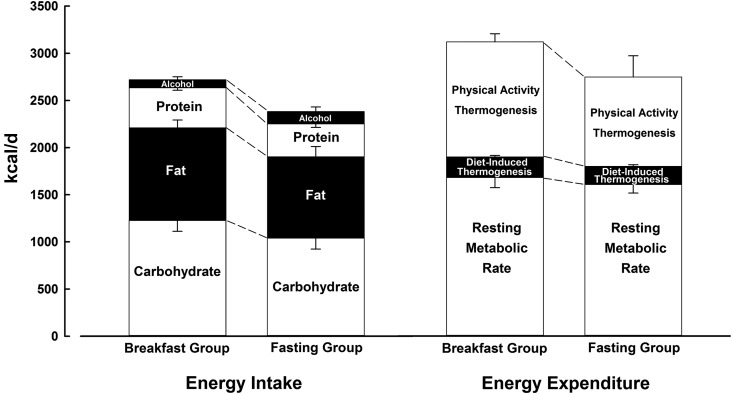
Components of energy balance under free-living conditions with either the ingestion of ≥700 kcal before 1100 daily (breakfast group) or abstinence from all energy-providing nutrients until at least 1200 daily (fasting group). Data are means with SE bars compared with use of independent *t* tests. Estimated energy intake values for comparison of relative differences between groups are the mean of the first (breakfast, *n* = 11: 2820 ± 595 kcal/d; fasting, *n* = 11: 2459 ± 780 kcal/d; *P* = 0.2) and last (breakfast, *n* = 11: 2618 ± 833 kcal/d; fasting, *n* = 11: 2303 ± 792 kcal/d; *P* = 0.4) weeks of intervention, for which a loss of data was caused by a loss of diet record. Resting metabolic rate values (breakfast group, *n* = 11; fasting group, *n* = 11) were data-recorded at follow-up, with 1 individual unable to complete the follow-up resting metabolic rate collection. Diet-induced thermogenesis values (breakfast group, *n* = 11; fasting group, *n* = 11) were estimated from reported energy intake, for which a loss of data was caused by a loss of diet record. Physical activity values are the mean of the first (breakfast, *n* = 9: 1204 ± 322 kcal/d; fasting, *n* = 10: 997 ± 887 kcal/d; *P* = 0.5) and last (breakfast, *n* = 9: 1238 ± 220 kcal/d; fasting, *n* = 10: 902 ± 543 kcal/d; *P* = 0.1) week of intervention.

**FIGURE 2:**
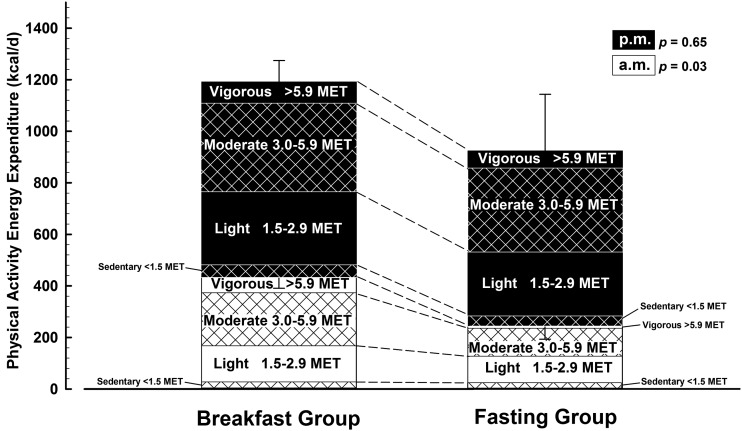
Physical activity thermogenesis under free-living conditions with either the ingestion of ≥700 kcal before 1100 daily (breakfast group, *n* = 9) or abstinence from all energy-providing nutrients until at least 1200 daily (fasting group, *n* = 10). Data are means with SE bars. *P* values represent the comparison between the 2 groups’ data for the mean of the 2 wk (1 and 6) of physical activity measurement with use of an independent *t* test. Missing data are the result of monitor failure or data of insufficient quality for analysis. Values are partitioned by the time of day and intensity of energy expenditure. MET, metabolic equivalent.

Physical activity thermogenesis is illustrated in **Figure 1** and partitioned in **Figure 2** to illustrate how and when this component of energy expenditure was accumulated. There was a significantly higher rate of physical activity thermogenesis in the breakfast group than the fasting group before 1200 daily (435 ± 132 kcal/d compared with 247 ± 171 kcal/d; *P* = 0.03) but no difference after 1200 (756 ± 135 kcal/d compared with 676 ± 540 kcal/d; *P* = 0.7), such that there was no consistent difference between groups over the entire day (1221 ± 261 kcal/d compared with 949 ± 709 kcal/d; *P* = 0.3). There were also no differences in the specific intensities of activity during any period of the day.

There were no differences between treatment groups in daily recordings of median (range) waking times [breakfast group: 0739 (0652–0840); fasting group: 0717 (0632–1046)] or sleeping times [breakfast group: 2255 (2227–0011); fasting group: 2312 (2155–0232)], such that mean sleep duration was similar between the breakfast group (501 ± 27 min/night) and the fasting group (486 ± 38 min/night).

#### Resting metabolic rate

Baseline assessments of the resting metabolic rate were not different between the breakfast and fasting groups (1679 ± 101 kcal/d compared with 1613 ± 79 kcal/d, respectively). The right-hand portion of Figure 1 presents follow-up data for these respective groups (1679 ± 106 kcal/d compared with 1605 ± 91 kcal/d). The resting metabolic rate was therefore stable within 8 kcal/d from baseline to follow-up, with no difference between groups in response to the intervention (*P* = 0.8).

#### Diet-induced thermogenesis

Based on established constants for the thermogenic effect of each macronutrient reportedly ingested according to food diaries (46), no difference in diet-induced thermogenesis was apparent between groups (Figure 1). In accordance with the relative similarity in energy intake and diet composition (see next section and Figure 1), diet-induced thermogenesis was 220 ± 55 kcal/d in the breakfast group and 193 ± 57 kcal/d in the fasting group (*P* = 0.3).

#### Energy intake

The breakfast group reported ingesting 2719 ± 683 kcal/d relative to 2381 ± 777 kcal/d reported by the fasting group (*P* = 0.3). The left-hand portion of Figure 1 illustrates that there were no differences in reported dietary macronutrient composition between the 2 groups, with all the data shown representing the mean over the intervention period (i.e., the mean of week 1 and week 6) given that both groups reported a similar small reduction in energy intake from the first to the last week (time effect: *F* = 4.9; *P* = 0.04), with the separate data for each period reported in the Figure 1 legend.

#### Energy balance regulatory hormones

Thyroid hormones that regulate resting metabolic rate were unresponsive to either treatment, with systemic concentrations of triiodothyronine (free-T3) and thyroxine (free-T4) closely matched between treatments at baseline and follow-up (**Table 2**). Similarly, a range of hormones implicated in the regulation of appetite and energy balance also did not differ in response between treatments. Table 2 presents fasted concentrations of leptin, total ghrelin, acylated ghrelin, peptide YY, active glucagon-like peptide-1, and adiponectin, all of which displayed no significant treatment × time interactions, although there was a tendency toward an interaction effect for leptin (*P* = 0.06).

**TABLE 2:** Metabolic/regulatory responses^1^

	All participants (*n* = 23)		Breakfast group (*n* = 11)		Fasting group (*n* = 12)	
	Baseline	Change from baseline	Baseline	Change from baseline	Baseline	Change from baseline
Regulatory hormones						
Triiodothyronine (free-T3),^2^ pg/mL	2.91 ± 0.47	−0.04 (−0.29, 0.21)	2.83 ± 0.34	0.10 (−0.38, 0.58)	2.99 ± 0.56	−0.15 (−0.45, 0.15)
Thyroxine (free-T4), ng/dL	1.20 ± 0.11	0.00 (−0.04, 0.04)	1.20 ± 0.11	0.02 (−0.03, 0.06)	1.20 ± 0.12	−0.01 (−0.08, 0.06)
Leptin,^2^ μg/L	31.3 ± 26.9	0.8 (−4.8, 6.3)	33.8 ± 35.7	6.7 (−2.6, 15.9)	27.1 ± 17.7	−3.5 (−10.4, 3.4)
Total ghrelin,^2^ pg/mL	416 ± 193	27 (−59, 113)	360 ± 177	−14 (−72, 45)	456 ± 202	57 (−95, 209)
Acylated ghrelin,^2^ pg/mL	63.2 ± 36.0	2.1 (−9.3, 13.5)	55.6 ± 34.5	−3.2 (−14.8, 8.3)	68.7 ± 37.7	6.0 (−13.4, 25.4)
Peptide YY,^2^ pg/mL	45.2 ± 26.9	4.2 (−2.2, 10.6)	38.9 ± 12.4	3.8 (−9.9, 17.6)	49.8 ± 33.7	4.5 (−3.1, 12.0)
Active glucagon-like peptide-1,^2^ pg/mL	4.51 ± 5.96	−0.55 (−2.03, 0.93)	2.64 ± 1.20	0.03 (−0.20, 0.27)	6.37 ± 3.58	−1.13 (−4.61, 2.35)
Adiponectin, mg/L	8.47 ± 4.03	−0.03 (−0.63, 0.58)	8.20 ± 4.73	−0.18 (−0.86, 0.50)	8.67 ± 3.68	0.08 (−0.94, 1.10)
Cardiovascular health						
Total cholesterol,^2^ mg/dL	205.8 ± 36.0	7.7 (1.5, 13.9)*	223.3 ± 30.6^#,†^	5.5 (−4.2, 15.2)	191.5 ± 34.8	9.5 (0.1, 19.0)
HDL cholesterol,^2^ mg/dL	48.5 ± 9.8	0.3 (−3.0, 3.5)	48.2 ± 9.3	1.0 (−2.3, 4.3)	48.7 ± 10.6	−0.4 (−6.2, 5.5)
LDL cholesterol,^2^^,^^3^ mg/dL	132.3 ± 31.7	8.4 (2.2, 14.7)*	151.1 ± 28.6^†^	5.7 (−2.6, 14.0)	118.7 ± 27.3	10.4 (0.4, 20.4)
Triacylglycerol,^2^ mg/dL	141.5 ± 79.0	−4.5 (−20.0, 11.0)	165.6 ± 98.0	−7.0 (−37.6, 23.6)	121.8 ± 56.8	−2.5 (−21.6, 16.6)
NEFA,^2^ mg/dL	13.88 ± 6.63	−1.57 (−4.10, 0.96)	15.46 ± 7.68	−3.70 (−8.46, 1.07)	12.73 ± 5.86	−0.03 (−3.09, 3.03)
IL-6,^2^ pg/mL	2.36 ± 2.60	−1.07 (−2.23, 0.09)	2.68 ± 3.66	−1.34 (−4.28, 1.61)	2.14 ± 1.63	−0.88 (−1.73, −0.03)
C-reactive protein,^2^ mg/L	2.60 ± 1.53	0.04 (−0.56, 0.63)	3.10 ± 2.05	−0.05 (−0.89, 0.80)	2.19 ± 1.03	0.11 (−0.87, 1.08)
Metabolic control						
Fasted glucose,^2^ mg/dL	97.9 ± 6.4	1.6 (−1.4, 4.5)	95.3 ± 5.3^†^	1.4 (−2.2, 5.1)	100.1 ± 6.6	1.7 (−3.6, 6.9)
Fasted insulin,^2^ μIU/mL	9.71 ± 4.42	−0.14 (−2.08, 1.80)	10.54 ± 5.88	0.39 (−3.44, 4.21)	8.96 ± 2.59	−0.62 (−2.74, 1.51)
HOMA-IR^4^	2.35 ± 1.02	0.02 (−0.50, 0.54)	2.46 ± 1.31	0.18 (−0.84, 1.19)	2.24 ± 0.72	−0.13 (−0.71, 0.45)
C-ISI Matsuda index^4^	3.80 ± 1.60	−0.00 (−0.54, 0.54)	3.78 ± 2.05	0.05 (−0.59, 0.70)	3.81 ± 1.09	−0.05 (−1.08, 0.97)
Insulin AUC glucose, mg · 120 min/dL	6400 ± 2118	−30 (−922, 863)	6882 ± 2449	−231 (−1745, 1283)	5917 ± 1735	171 (−1132, 1475)

1 Data are means ± SDs at baseline, with Δs and 95% CIs for the response within each group. *Significant response over time (*P* ≤ 0.05), ^#^significant difference between groups at baseline (*P* = 0.03), and ^†^main effect of group (*P* ≤ 0.05) as examined by 2-factor ANOVA. There were no significant interactions for any of the variables. C-ISI, composite insulin sensitivity index; DXA, dual-energy X-ray absorptiometry; NEFA, nonesterified fatty acid; OGTT, oral-glucose-tolerance test.

2 SI conversions: cholesterols × 0.0259 = mmol/L; triacylglycerol × 0.0113 = mmol/L; NEFA × 0.0355 = mmol/L; IL-6 × 0.131 = IU/mL; C-reactive protein × 9.524= nmol/L; glucose × 0.0555 = mmol/L; insulin × 6.0 = pmol/L; free-T3 × 1.54 = pmol/L; free-T4 × 12.87 = pmol/L; leptin × 0.0625 = nmol/L; ghrelin × 0.296 = pmol/L; peptide YY × 4.31 = pmol/L; and active glucagon-like peptide 1 × 0.303 = pmol/L.

3 Calculated with use of the Friedwald equation (LDL cholesterol = total cholesterol − HDL cholesterol − (triacylglycerol/2.2)].

4 HOMA-IR = (fasted insulin in μIU/m^L^ × fasted glucose in mmol/L)/22.5; C-ISI Matsuda index = 10,000/SQRT (fasted glucose in mg/dL × fasted insulin in μIU/mL) × (mean glucose over 120 min OGTT mg/dL × mean insulin over 120 min OGTT in μIU/mL).

### Health risk factors

#### Anthropometric factors

Body mass increased from pre- to postintervention (0.6 kg; 95% CI: 0.1, 1.1 kg) when all participants from both groups were considered (*F* = 6.3; *P* = 0.02). The absolute change was greater in the breakfast group (1.0 kg; 95% CI: 0.2, 1.7 kg) compared with the fasting group (0.2 kg; 95% CI: −0.5, 1.0 kg) but with no interaction between treatment and time (*F* = 2.2; *P* = 0.15). None of the measures of body composition was differently affected by the interventions (Table 1).

#### Cardiovascular health

None of the cardiovascular disease risk factors presented in Table 2 responded differently between groups to the intervention (all *P* > 0.1). There was an increase in total and LDL cholesterol concentrations across both groups from pre- to postintervention (both *P* < 0.03).

#### Metabolic control

**FIGURE 3:**
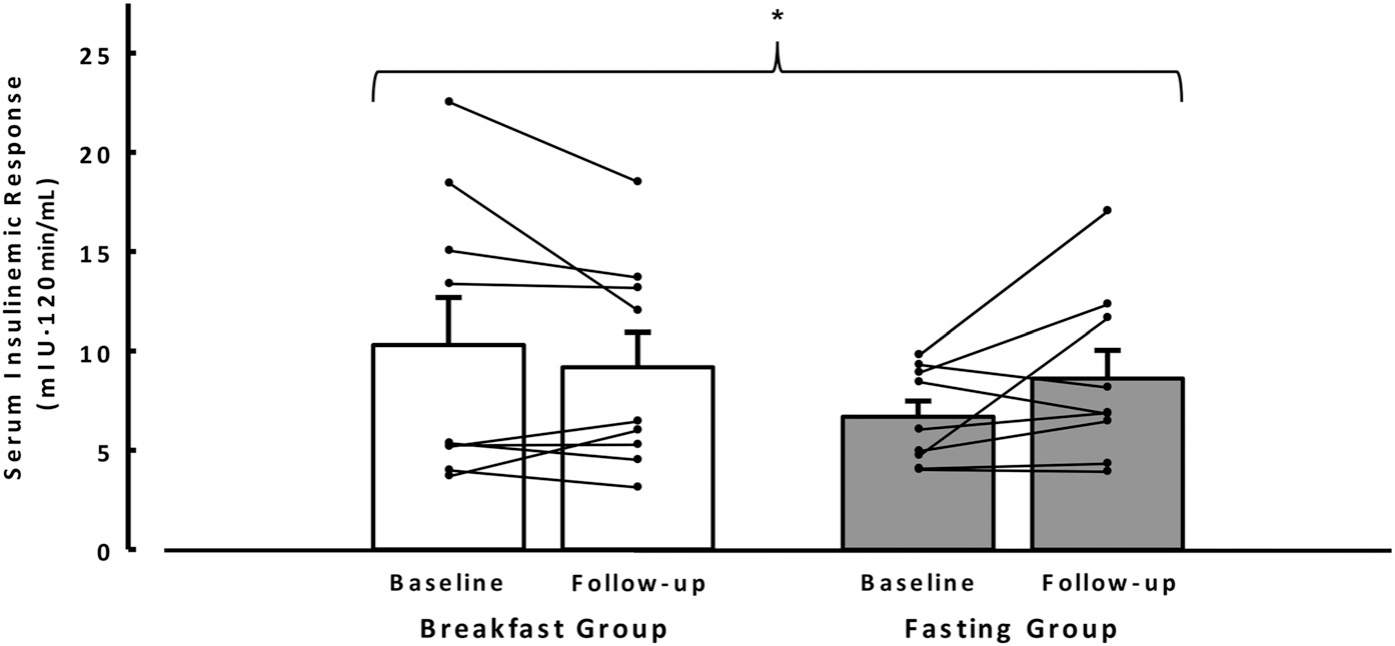
Insulinemic responses to the oral-glucose-tolerance test measured at baseline and after 6 wk (follow-up) of ingestion of ≥700 kcal before 1100 daily (breakfast group, *n* = 9) or abstinence from all energy-providing nutrients until at least 1200 daily (fasting group, *n* = 9), for which missing data resulted from cannula failure. Bars are mean incremental AUCs with SE bars, and lines are paired individual responses from baseline to follow-up. There was no main effect of treatment or time detected by 2-factor ANOVA. *Treatment × time interaction (*F* = 4.7; *P* = 0.05) for the insulinemic response to the oral-glucose-tolerance test.

Fasting plasma glucose and serum insulin concentrations did not change over time (both *P* > 0.3), with no evidence of a treatment × time interaction (both *P* > 0.6) (Table 2). Both Matsuda and HOMA indexes of insulin sensitivity were not different over time (*P* > 0.3), with no evidence of an interaction (*P* > 0.5). Glycemic response to the OGTT was unaffected by either intervention (*P* > 0.17). There were no main effects of treatment or time (i.e., baseline follow-up) on insulin incremental AUC (both *P* > 0.3), but there was a significant treatment × time interaction with an increase in the fasting group from baseline relative to a decrease in the breakfast group from pre- to postintervention (*P* = 0.05) (**Figure 3**).

**FIGURE 4:**
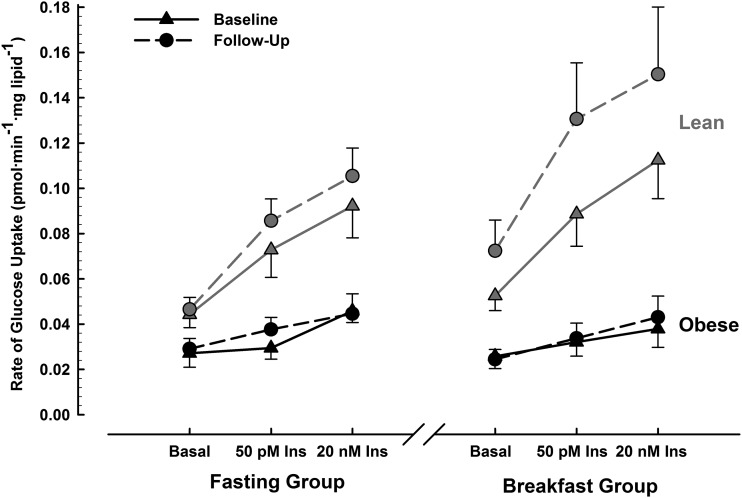
Rates of [U-^14^C]d-glucose uptake in adipocytes under basal, physiologic (50 pmol/L insulin) and supraphysiologic (20 nmol/L insulin) conditions, measured at baseline and after 6 wk of ingestion of ≥700 kcal before 1100 daily (breakfast group, *n* = 9) or abstinence from all energy-providing nutrients until at least 1200 daily (fasting group, *n* = 10), for which missing data resulted from insufficient adipose tissue obtained from the biopsy. Data are means with SE bars. Three-factor ANOVA (treatment × time × insulin) reveals a significant main effect of insulin (*F* = 23; *P* < 0.001) but with no significant main effects of treatment, time, or any interaction of these factors (all *P* > 0.05). Lean data displayed in gray have previously been published (15) and are included to provide a frame of reference for these obese data. Ins, insulin.

The rates of adipose tissue-specific glucose uptake obtained in these individuals is displayed in **Figure 4**. There was a main effect of insulin (*F* = 23; *P* < 0.001) but no main effects of treatment, time, or any interaction of these effects (all *P >* 0.05). For context, the obese population reported herein had much lower insulin-stimulated glucose uptake than the lean population we reported previously (15), particularly with maximal (supraphysiologic) insulin stimulation. Because of these very low absolute rates of glucose uptake/responsiveness to insulin, the index of adipose tissue insulin sensitivity we previously calculated in lean individuals could not be used for these data.

Subcutaneous glucose was monitored continuously at 5-min intervals throughout the first and last week of intervention. Mean and peak glucose concentrations from waking until 1200 and from 1200 until sleep were similar between groups at baseline and over time, and there was thus no difference in response to the intervention between groups. The nocturnal glycemic control data from the continuous glucose monitoring system in 5 individuals contained implausibly stable periods at the lower extremity of the physiologic/detectable range (2.2–2.5 mmol/L), although only for <10% of measurements from these individuals. When this occurred for more than 4 consecutive measurements (>15 min), these data were removed (this did not affect the conclusions drawn). Nocturnal (sleeping) peak values did not differ between groups (breakfast group: 7.0 ± 1.4; fasting group: 7.3 ± 1.1 mmol/L), but there was a main effect of treatment reflecting lower mean overnight glucose concentrations in the breakfast group than the fasting group across the first and last week of intervention (breakfast group: 4.9 ± 0.5; fasting group: 5.4 ± 0.6 mmol/L; *F* = 4.4; *P* = 0.05).

The CV is the preferred method for expressing glucose variability (accumulated hyper- and hypoglycemic episodes) when continuously monitored glucose data are available for individual patients (47). This measure did not reveal any differences between groups over time or in response to the intervention between groups from waking until 1200 and from 1200 until sleep or nocturnally. Equally, none of these variability, mean, or peak glucose values varied over the full 24-h period.

## DISCUSSION

This is the first experiment to our knowledge to measure all components of energy balance and selected markers of metabolic control and cardiovascular disease risk in response to daily morning fasting compared with breakfast consumption for 6 wk in healthy obese individuals. Four key findings from this study were that there was *1*) no difference in reported total energy intake between interventions, indicative that those fasting during the morning at least partially compensated for the ≥700 kcal deficit imposed; *2*) lower physical activity thermogenesis in those fasting before 1200 than in those who consumed breakfast; *3*) similar blood lipid, appetite regulatory hormone, and C-reactive protein responses to the intervention between groups but with a decreased insulinemic response to an OGTT in those consuming breakfast relative to an increase in those extending their fast; and *4*) no evidence that the omission of breakfast had any effect on body weight.

Energy intake in those who habitually skip breakfast has been reported in cross-sectional studies to be similar (31, 48) and lower than those regularly consuming breakfast (6, 49). Experimental evidence in individuals omitting breakfast without a prescribed daily energy deficit has also produced conflicting results, with greater (14), similar (13) and lower intake (7) in those missing breakfast. Our previous study examined the same interventions as reported herein in lean individuals (15). In contrast to the current data, that experiment revealed that individuals assigned to morning fasting had considerably lower energy intake than those consuming breakfast, with minimal dietary compensation throughout the rest of the day.

When attempting to reconcile our results with the extant literature, it is possible differences in the extent to which breakfasts were prescribed may account for some of the discrepancy. Two of the studies previously mentioned allowed ad libitum intake of breakfast and foods throughout the day (7, 13), whereas Farshchi et al. (14) prescribed identical foods consumed at 0800 in the breakfast condition or delayed until 1100 in the no-breakfast condition, with scheduled meal patterns/composition. In our experiment, individuals in the breakfast group were required to consume ≥700 kcal by 1100 in the breakfast condition. The impact of differing energy intake at breakfast during free living has not been well-investigated, although work by Martin et al. (18) established that free-living energy intake in lean men was greater when consuming a 700-kcal than a 100-kcal breakfast. This is contrary to the current data and therefore may indicate a modifying effect of weight status on energy intake during breakfast/morning fasting.

Reeves et al. (7) reported lower energy intake with morning fasting in lean and overweight/obese individuals who in a crossover design both ate and skipped breakfast for 7 d. However, this was a pooled effect, and the difference in overweight/obese individuals (∼60 kcal) was less pronounced than in lean individuals (∼265 kcal). This finding is consistent with the interpretation that obese individuals display greater compensation for a morning caloric deficit than lean counterparts. Reasons for greater reported dietary compensation with increasing adiposity are not immediately apparent, although it should be noted that underreporting of energy intake is greater in the obese (50). Despite this, there is no reason to suspect that underreporting should occur to a greater extent in either experimental (i.e., breakfast or fasting) group (51).

In this study, obese individuals undertaking daily morning fasting displayed lower physical activity expenditure before 1200. This finding verifies our earlier observation in lean individuals that physical activity energy expenditure is specifically most affected during the period in which energy intake is restricted (15), a finding also consistent with evidence of lower physical activity in the morning in adolescents that skip breakfast more frequently (52). The precise reasons for lesser morning physical activity expenditure when fasting remain to be established but may be related to perceptions of lethargy and/or expectations relating to physical activity readiness. In the future it would be informative to use subjective markers of energy throughout the day [e.g., the subjective vitality scale (53)] with breakfast and fasting regimens. In contrast to our previous findings in lean individuals (15) in which a greater overall effect of breakfast was apparent (442 kcal/d; 95% CI: 34, 851), total daily physical activity thermogenesis was not significantly higher when consuming breakfast among this obese population (272 kcal/d; 95% CI: −254, 798). This study was powered to detect an effect sufficient to produce a net negative energy balance, and because the magnitude of difference was far less than the energy directly provided by breakfast, it was not deemed statistically significant. Although even a small increase in physical activity may be associated with positive health outcomes independent of energy balance, the difference in energy expenditure we previously reported in lean individuals was ∼60% greater than reported herein, which mirrors the lack of difference in overall energy intake in obese individuals. Therefore, responses of individual components of energy balance to fasting may be generally less marked with greater adiposity.

The measurement of greater physical activity energy expenditure during the morning when breakfast is consumed is unlikely to be attributable simply to tachycardia after feeding because 480 kcal of mixed macronutrients resulted in elevations of only ∼5 beats/min in lean and obese women (54). Moreover, even elevations in heart rate alone cannot account for the differences in energy expenditure (particularly at light intensity) measured in this study because the branched model used by the Actiheart device weights energy expenditure estimates heavily toward accelerometry unless the heart rate is substantially above rest. In addition, the reported energy intake of the fasting group from midday onward was ∼550 kcal greater than that of the breakfast group, further supporting that energy expenditure measured with use of this device is not predicated on an acutely increased heart rate after meals. Although it is possible this effect on physical activity may have been caused by the behavior of eating, cooking activities span the range of 2.5–3.5 metabolic equivalents (55), so the potential energetic contribution would have been small (∼50–75 kcal), even assuming a generous 30-min food preparation period substituted for complete rest when fasting.

Blood lipid concentrations were not different between interventions in this cohort. This differs from the findings of Farshchi et al. (14), who reported increased systemic LDL and total cholesterol concentrations after 2 wk of breakfast omission in lean women. That effect was attributed to reduced stimulation of hydroxyl methyl glutaryl-Co-A reductase by insulin given that the insulinemic response to a mixed-meal challenge was lower after breakfast consumption. Similar to that study, herein we report an interaction for the insulin AUC in response to the OGTT such that insulinemia decreased in those consuming breakfast relative to an increase in those extending their fast (indicating reduced insulin sensitivity in those that fasted). However, HOMA-IR and Matsuda composite insulin sensitivity index measures of insulin sensitivity and insulin-stimulated glucose uptake into isolated adipocytes were unchanged. It remains to be established whether these examples of specific responses to meal challenges will translate into ecologically relevant benefits in response to repeated mixed-meal ingestion.

In view of the public perception that breakfast consumption facilitates weight management (1), it is paradoxical that 10 of the 11 individuals in the breakfast group gained weight (1.0 kg; 95% CI: 0.2, 1.7 kg). Although in the absence of a significant treatment × time interaction for body mass it cannot be concluded that daily breakfast causes weight gain, it is clear that ensuring the overnight fast is broken upon waking does not cause weight loss in a free-living setting. This interpretation is consistent with a large 16-wk intervention that recommended breakfast consumption or fasting until 1100 daily in overweight/obese individuals (9), so the conclusion that breakfast per se is not causally related to weight change is not specific to the precise nature of our intervention. Therefore, caution should be exercised when considering recommendations that advocate breakfast in general for the purpose of weight loss in obese individuals. However, it remains to be established whether breakfasts of specific composition could better target weight management, but data such as reported herein may provide the understanding necessary to target mechanisms that would best allow this (e.g., by enhancing/limiting compensatory responses to breakfast/fasting, respectively).

To our knowledge, this study provides the first evidence from a randomized controlled trial that sustained daily breakfast omission affects some indexes of insulin sensitivity in healthy obese individuals and adds to the body of evidence that breakfast consumption can maintain insulin sensitivity (14) and glycemic control (15) in lean individuals and that greater breakfast quantity can improve glycemic control in type 2 diabetes (10, 56). It is therefore important to recognize that randomized controlled trials in a variety of populations have demonstrated health benefits of breakfast consumption beyond mere weight management. With this in mind, there is a need for the popular media, health professionals, and public health messages to discriminate between the effects (or potential lack thereof) of breakfast consumption on body weight and the substantiated role for regular breakfast consumption in influencing other aspects of health.

In summary, we conclude that neither overall energy intake nor physical activity is different in obese individuals fasting during the morning or consuming a daily breakfast for 6 wk. However, differences in the distribution of physical activity throughout the day were apparent, with lower physical activity during the morning in response to fasting. Resting metabolic rate and blood lipid profiles were not differently affected by breakfast or fasting, although there was some evidence of breakfast omission reducing insulin sensitivity. Furthermore, regular daily breakfast did not facilitate weight loss.
